# Supplementary figures and images for: Downy Mildew effector HaRxL21 interacts with the transcriptional repressor TOPLESS to promote pathogen susceptibility
Source: PLoS Pathog. 2020 Aug 12;16(8):e1008835. doi: 10.1371/journal.ppat.1008835 (PMC7446885; doi:10.1371/journal.ppat.1008835)

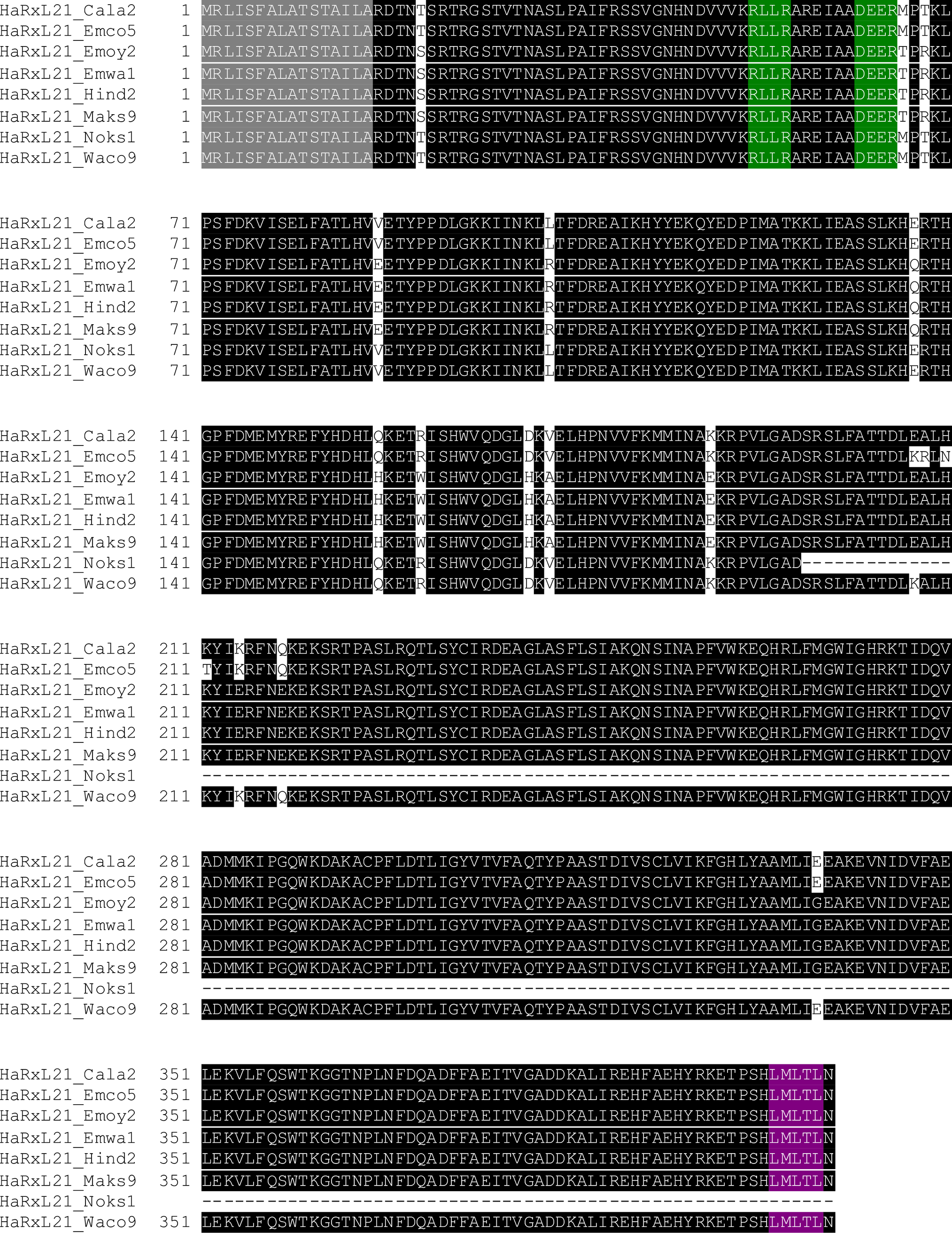

Supplement: S1 Fig — Sequences were obtained from Asai et al., 2018 (Cala2, Emco5, Emoy2, Emwa1, Hind2, Maks9 and Waco9) and BioProject PRJNA298674 (Noks1). Multiple sequence alignment was performed using T-coffee (http://tcoffee.crg.cat/apps/tcoffee/do:mcoffee). Sites of amino acid substitution between alleles are highlighted in white. Predicted signal peptide is shown in grey. The RxLR-DEER motif (green) and EAR motif (magenta) are conserved across alleles except Noks1. (TIF) [file ppat.1008835.s002.tif]

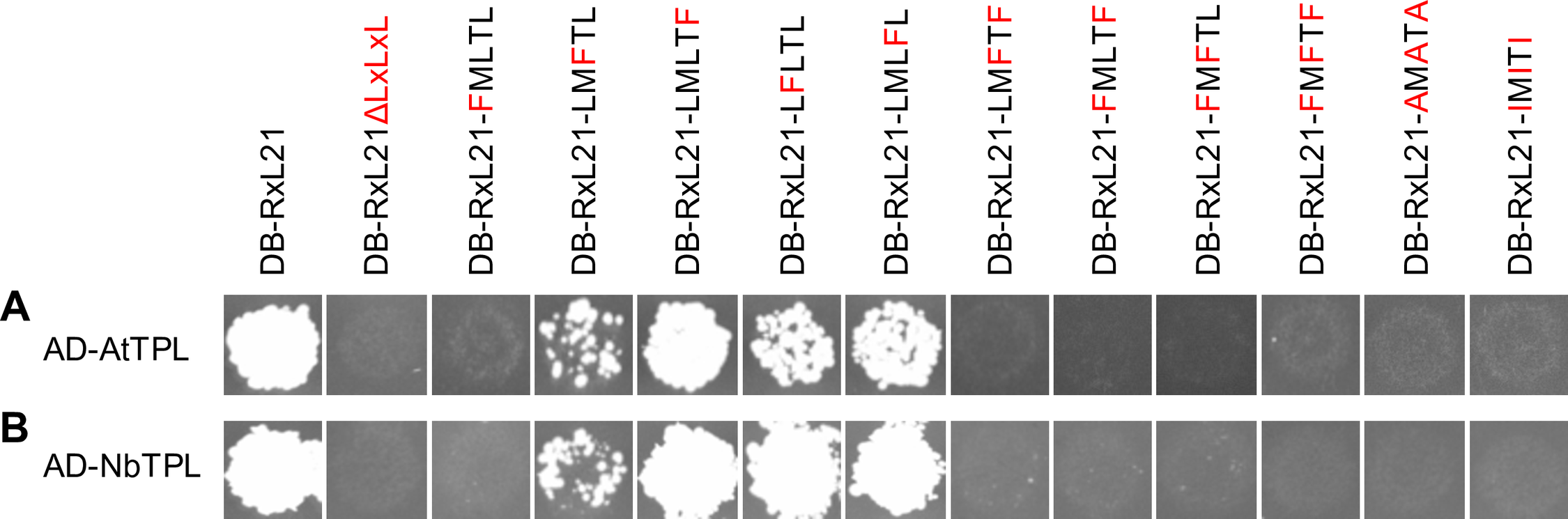

Supplement: S2 Fig — Leu residues in the EAR (LxLxL) motif were mutated to Phenylalanine (F), Alanine (A) and Isoleucine (I). Amino acids that differ from WT are indicated in Red. AD; GAL4 activation domain. DB; GAL4 DNA binding domain. (A) Interaction was tested against TPL from Arabidopsis (AtTPL). (B) Interaction was tested with TPL from N. benthamiana (NbTPL). Growth on media lacking Leu, Trp and His is shown, indicating successful mating and activation of the GAL1::HIS3 reporter gene due to interaction. All combinations tested showed growth on media lacking only Leu and Trp indicating successful mating (see S1 Data). The experiment was repeated on multiple plates with similar results. (TIF) [file ppat.1008835.s003.tif]

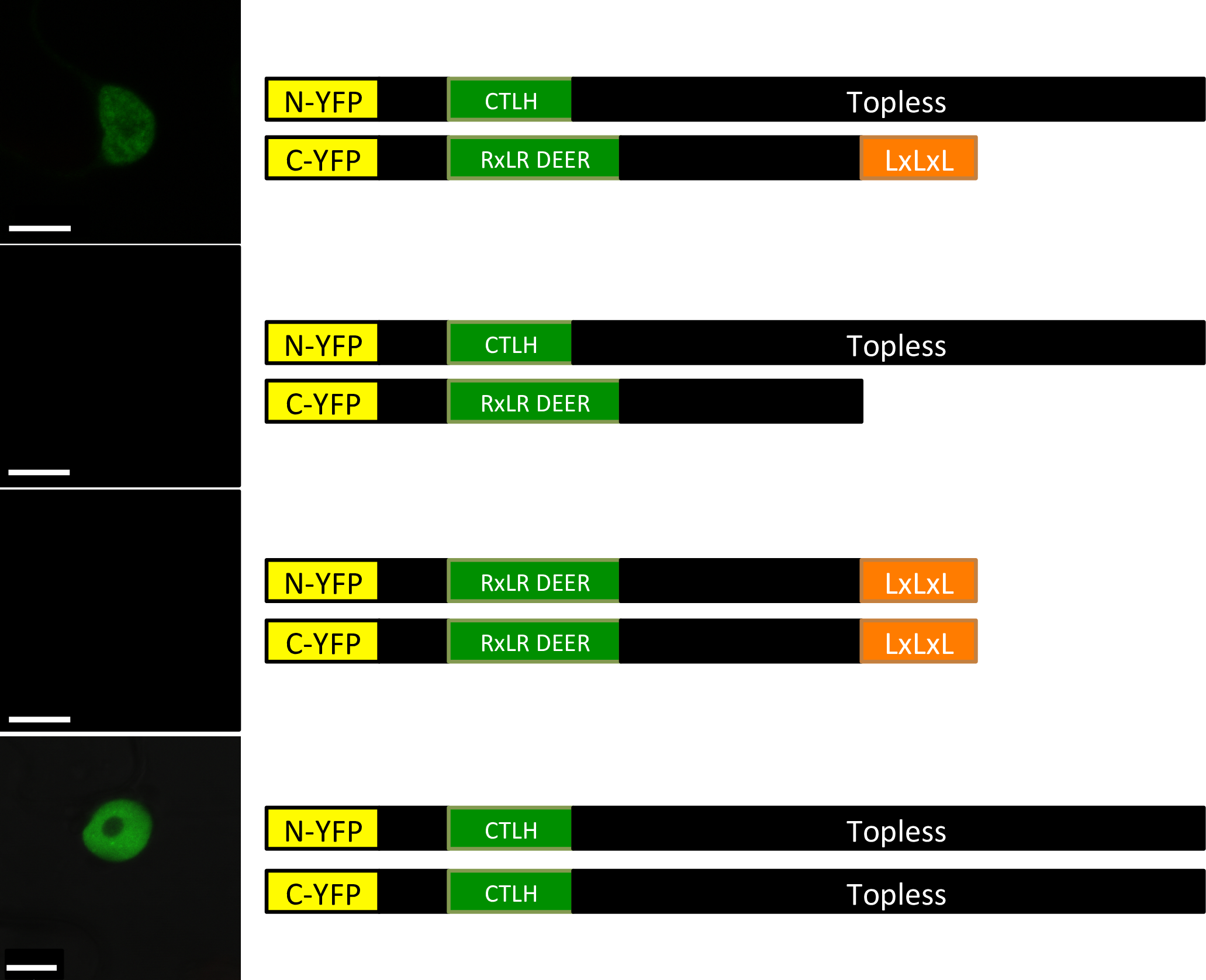

Supplement: S3 Fig — The BiFC assay was conducted transiently in N. benthamiana epidermal cells by co-agroinfiltration of TPL(YFPN) and RxL21(YFPC), TPL (YFPN) and RxL21ΔEAR(YFPC), RxL21(YFPN) and RxL21(YFPC), TPL(YFPN) and TPL(YFPC). Infiltrated tissues were imaged at 48 hpi by confocal scanning laser microscopy for YFP fluorescence. The interaction between TPL and RxL21 was lost with deletion of the EAR motif. RxL21 does not appear to form dimers unlike TPL where strong fluorescence was observed in the nucleus. Scale bar = 10 μm. (TIF) [file ppat.1008835.s004.tif]

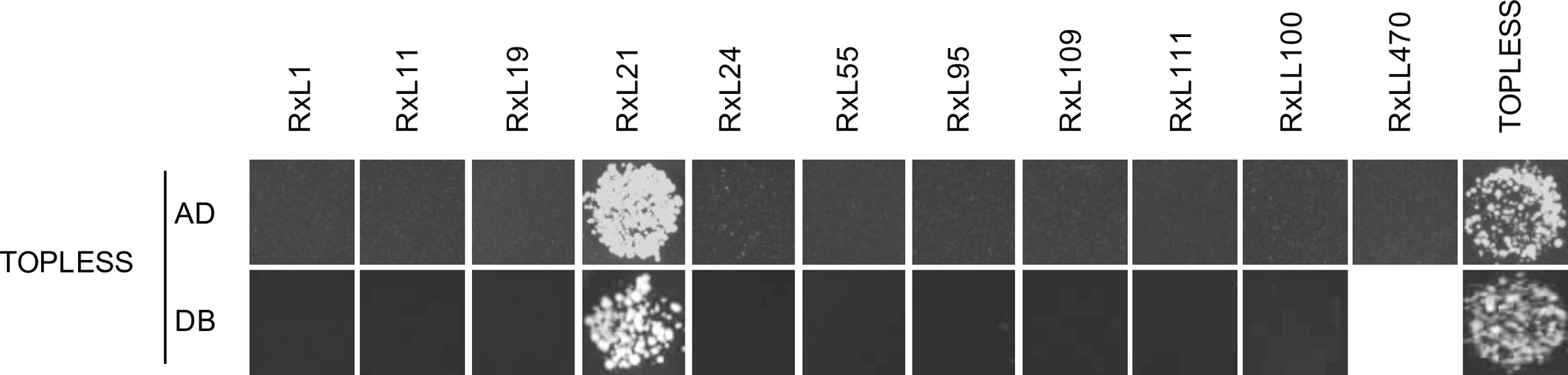

Supplement: S4 Fig — Y2H was performed using activation domain (AD)-TPL and DNA-binding domain (DB)-Effector constructs (top) and DB-TPL with AD-Effector constructs (bottom row). TPL interaction with HaRxL21 and TPL dimerisation were used as positive controls. Protein-protein interaction is shown by growth (indicating GAL1::HIS3 reporter gene activation) on SC media lacking Leucine, Tryptophan and Histidine. All combinations tested also showed growth on–LW media indicating successful mating (see S1 Data). The experiment was repeated on multiple plates with similar results. (TIF) [file ppat.1008835.s005.tif]

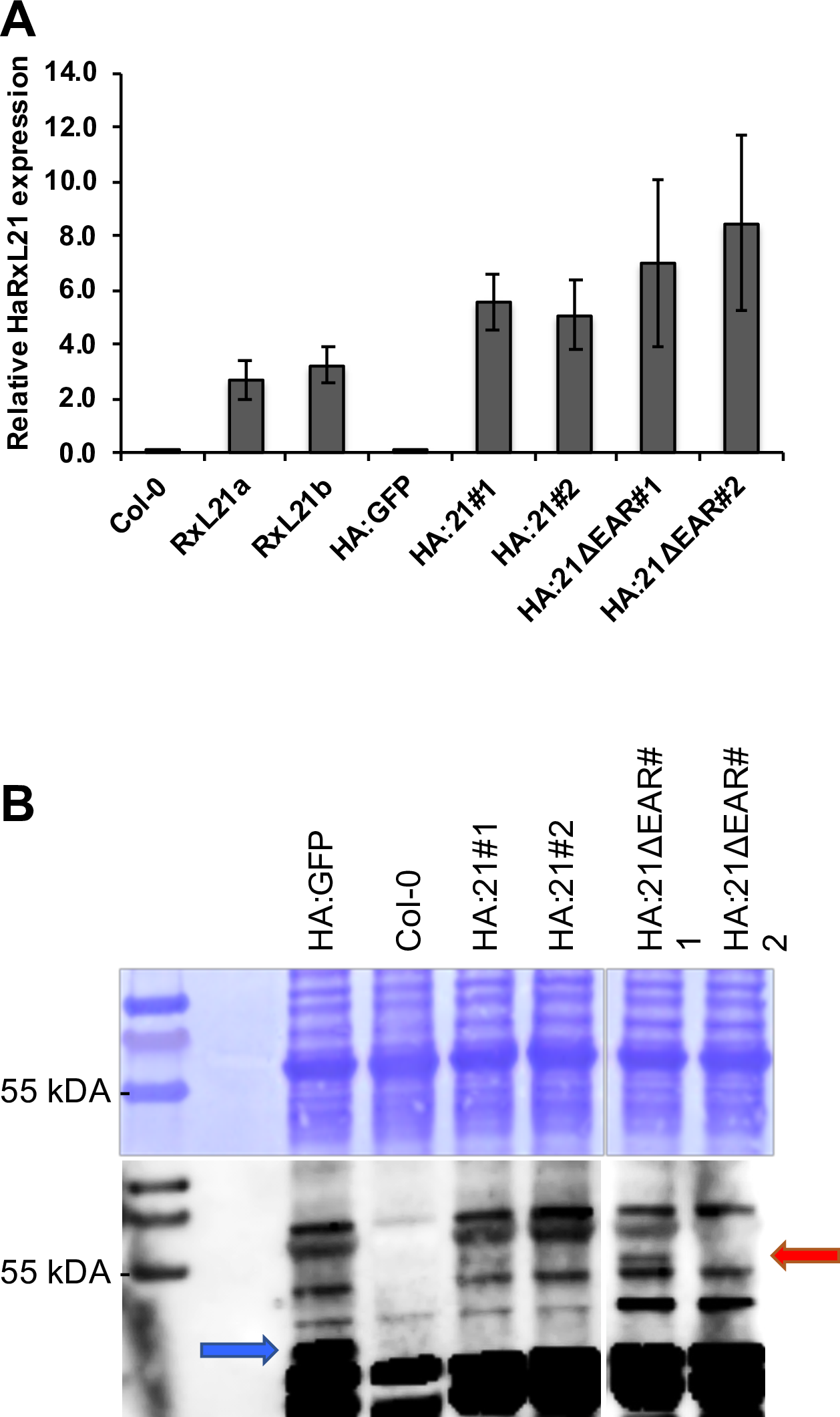

Supplement: S5 Fig — (A) Expression of RxL21 by quantitative RT-PCR relative to AtAct2 and UBQ5. Error bars show variance between technical replicates. Previously characterized 35S lines RxL21a/b were included for comparison. (B) Upper: Coomassie staining, lower: western blot using anti-HA. GFP and RxL21 bands are indicated by blue and red arrows respectively. (TIF) [file ppat.1008835.s006.tif]

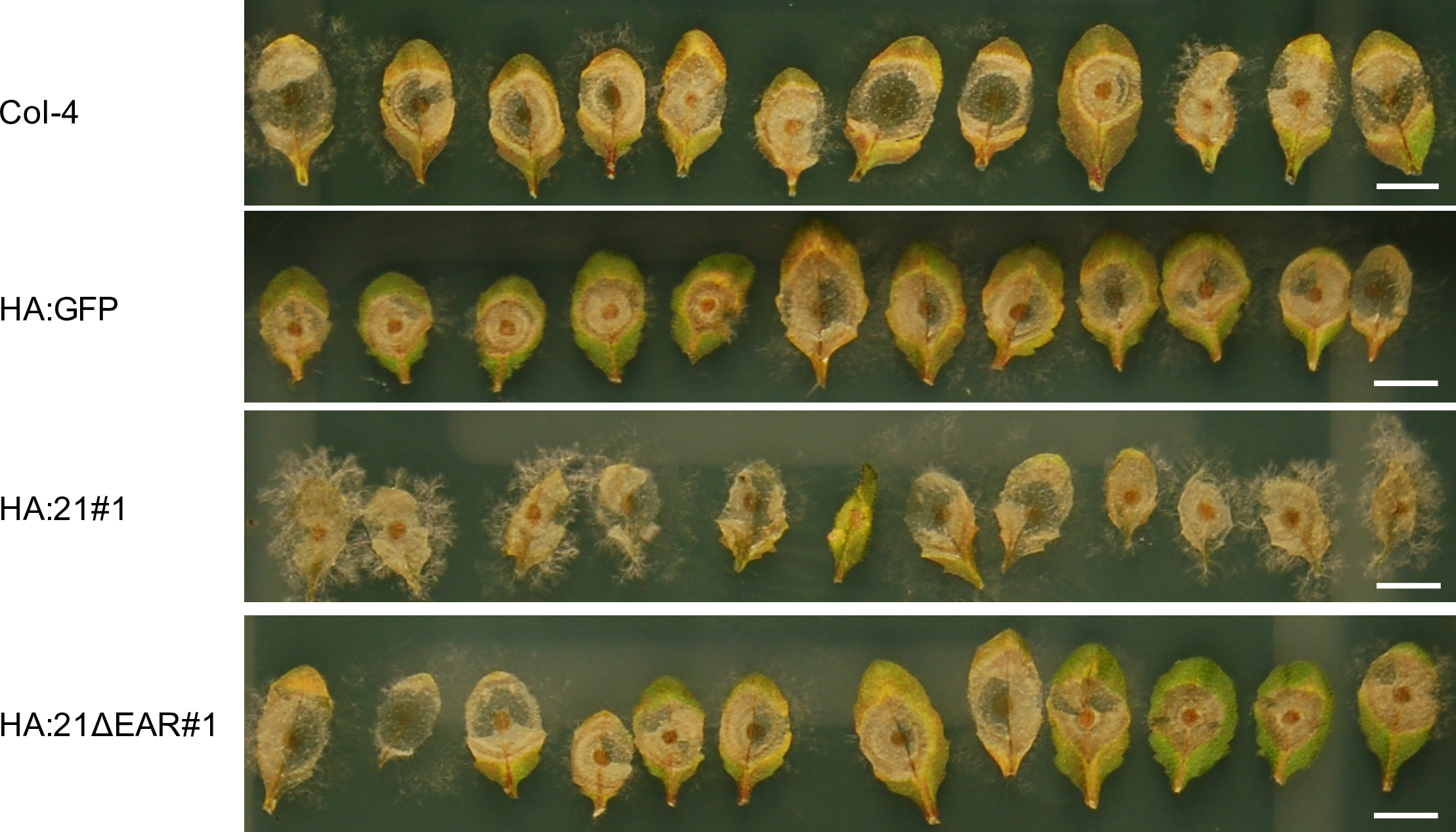

Supplement: S6 Fig — Photos taken 96 h post-infection with Botrytis cinerea. HA:21#1 appears to show more sporulation compared to Col-4, 35S::HA::GFP (HA:GFP) and HA:21ΔEAR#1. Scale bar is 1 cm. (TIF) [file ppat.1008835.s007.tif]

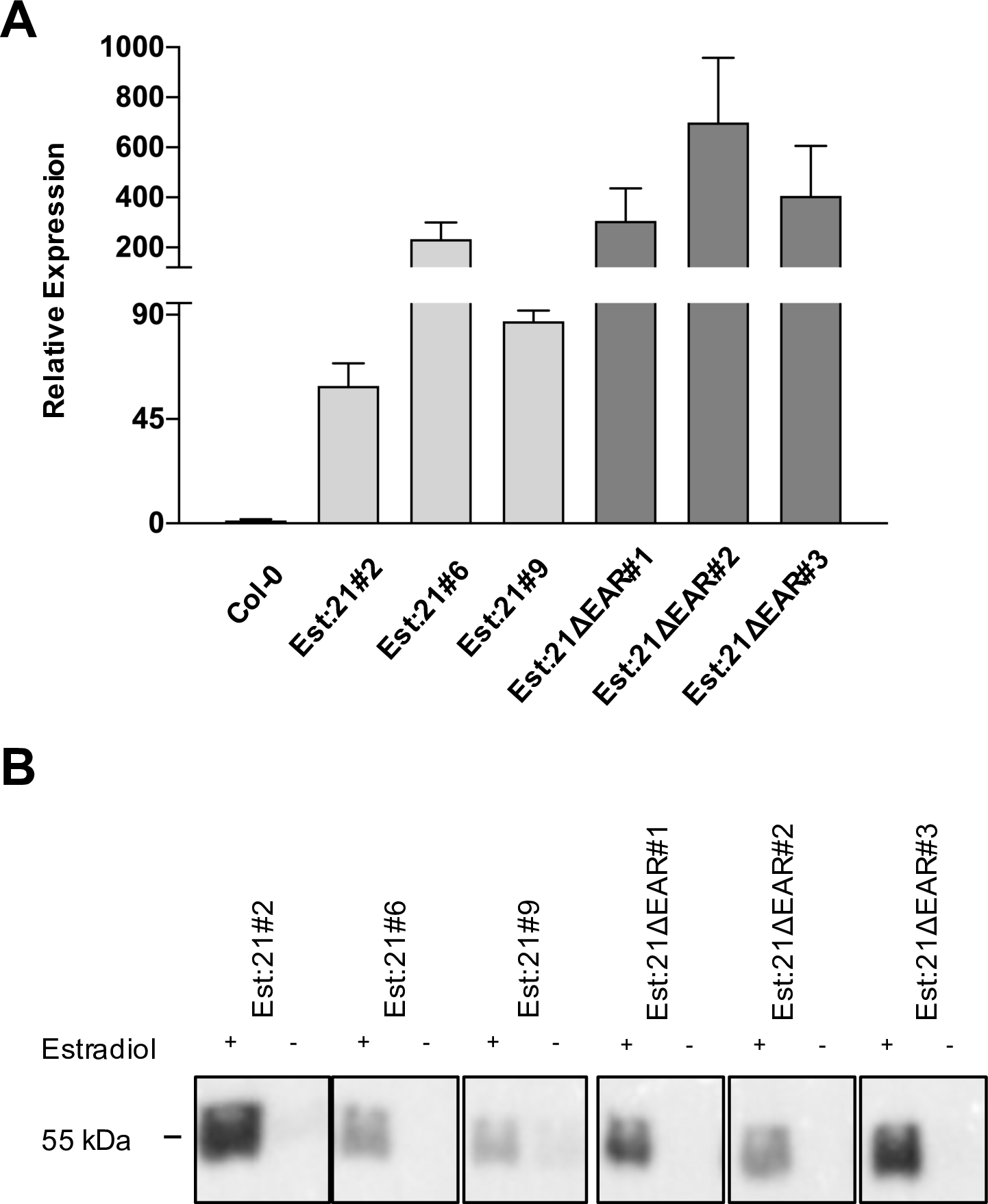

Supplement: S7 Fig — (A) Relative expression of RxL21 in Arabidopsis plants expressing myc::RxL21 and myc::RxL21ΔEAR (under an estradiol (Est) inducible promoter) was determined by quantitative RT-PCR 18 h after induction with 30 μM estradiol. Expression levels were normalised to Arabidopsis tubulin 4. Error bars show standard error between 3 biological replicates. (B) Anti Myc immunoblot showing Est-inducible expression of RxL21 and RxL21ΔEAR in Arabidopsis lines. Samples were taken 18 h after induction with 30 μM estradiol. (TIF) [file ppat.1008835.s008.tif]

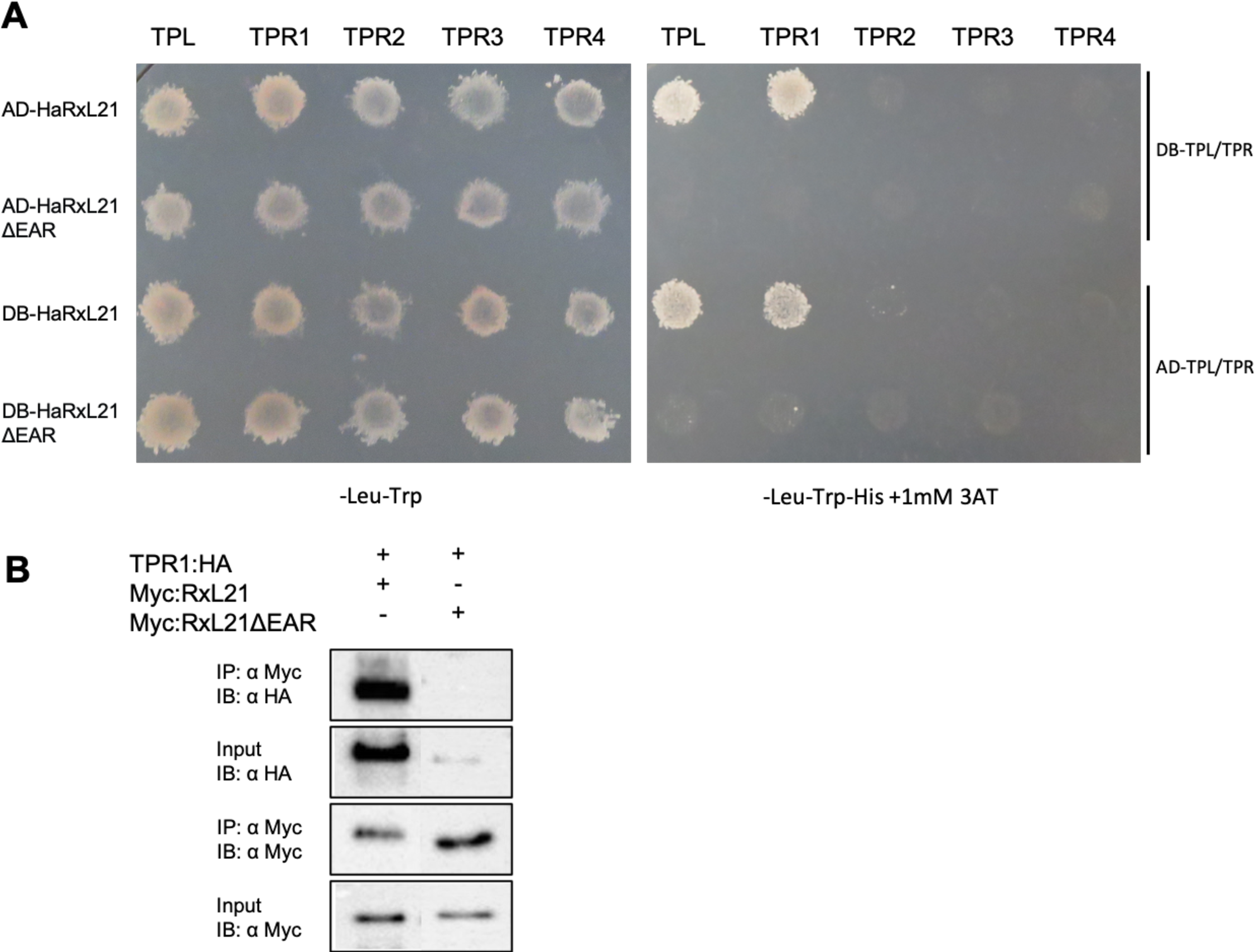

Supplement: S8 Fig — (A) RxL21 interacts with TPL and TPR1 by Y2H, indicated by growth on media lacking Leucine (Leu), Tryptophan (Trp) and Histidine (His). Growth on–Leu-Trp media indicates successful mating. Y2H was repeated in both directions, with both RxL21 and TPRs fused to AD; activation domain and DB; DNA binding domain. Y2H was repeated on multiple plates with similar results. (B) TPR1 interacts with RxL21 in planta. TPR1:HA with Myc:RxL21 or Myc:RxL21ΔEAR were transiently expressed in N. benthamiana leaves and harvested after 48 h. RxL21 expression was induced by 30μM β-estradiol 24 h prior to harvesting. α-myc beads were used for immunoprecipitations (IP). HA antibody was used to detect TPR1 immunoblots (IB) and α-myc antibody was used to detect RxL21 and RxL21ΔEAR. (TIF) [file ppat.1008835.s009.tif]

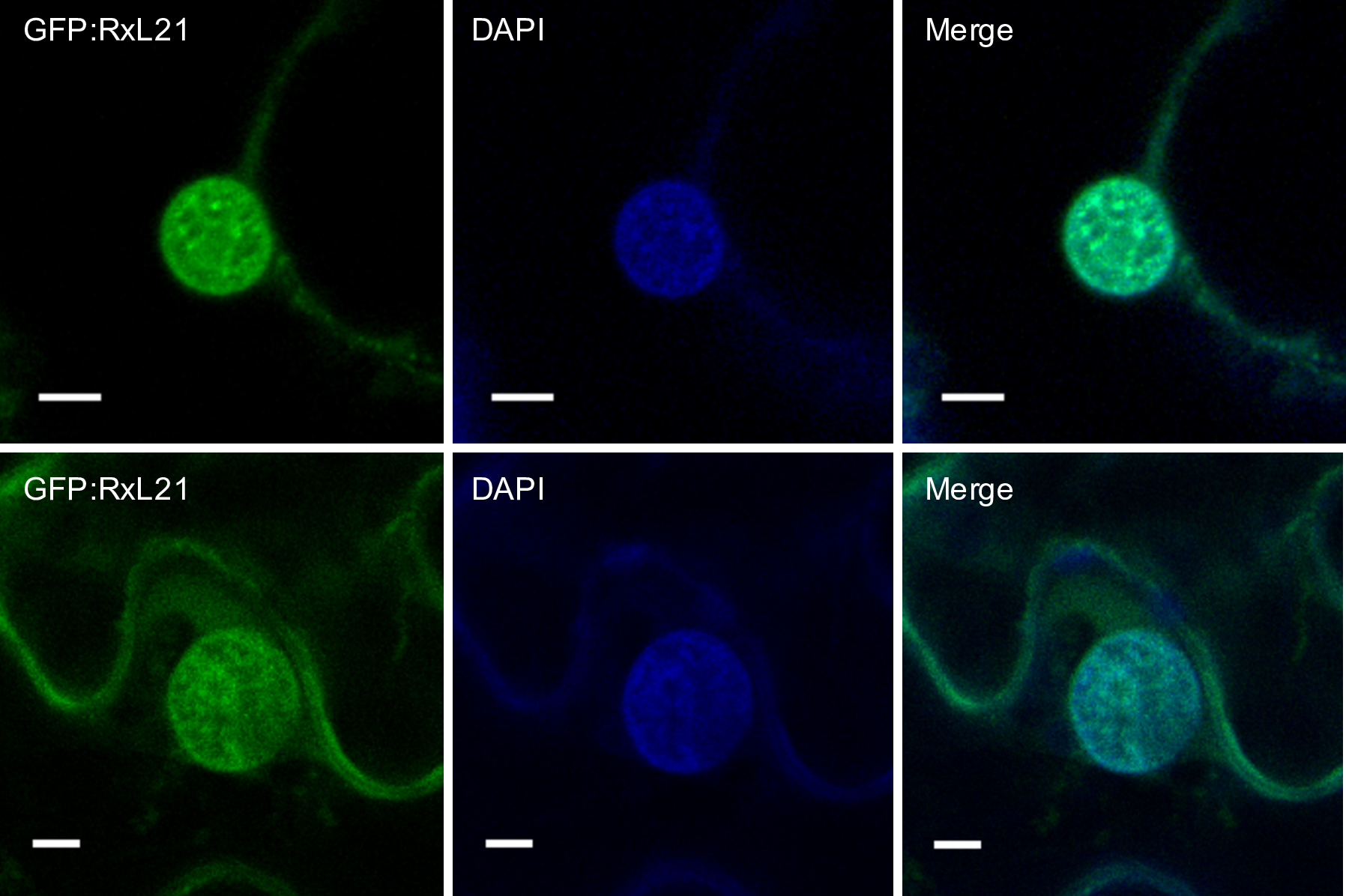

Supplement: S9 Fig — Transient expression of GFP:RxL21 using Agrobacterium mediated transformation of N. benthamiana. Immediately prior to imaging, leaves were stained by infiltration with DAPI (4′,6-diamidino-2-phenylindole). Scale bars are 5 μm. (TIF) [file ppat.1008835.s010.tif]

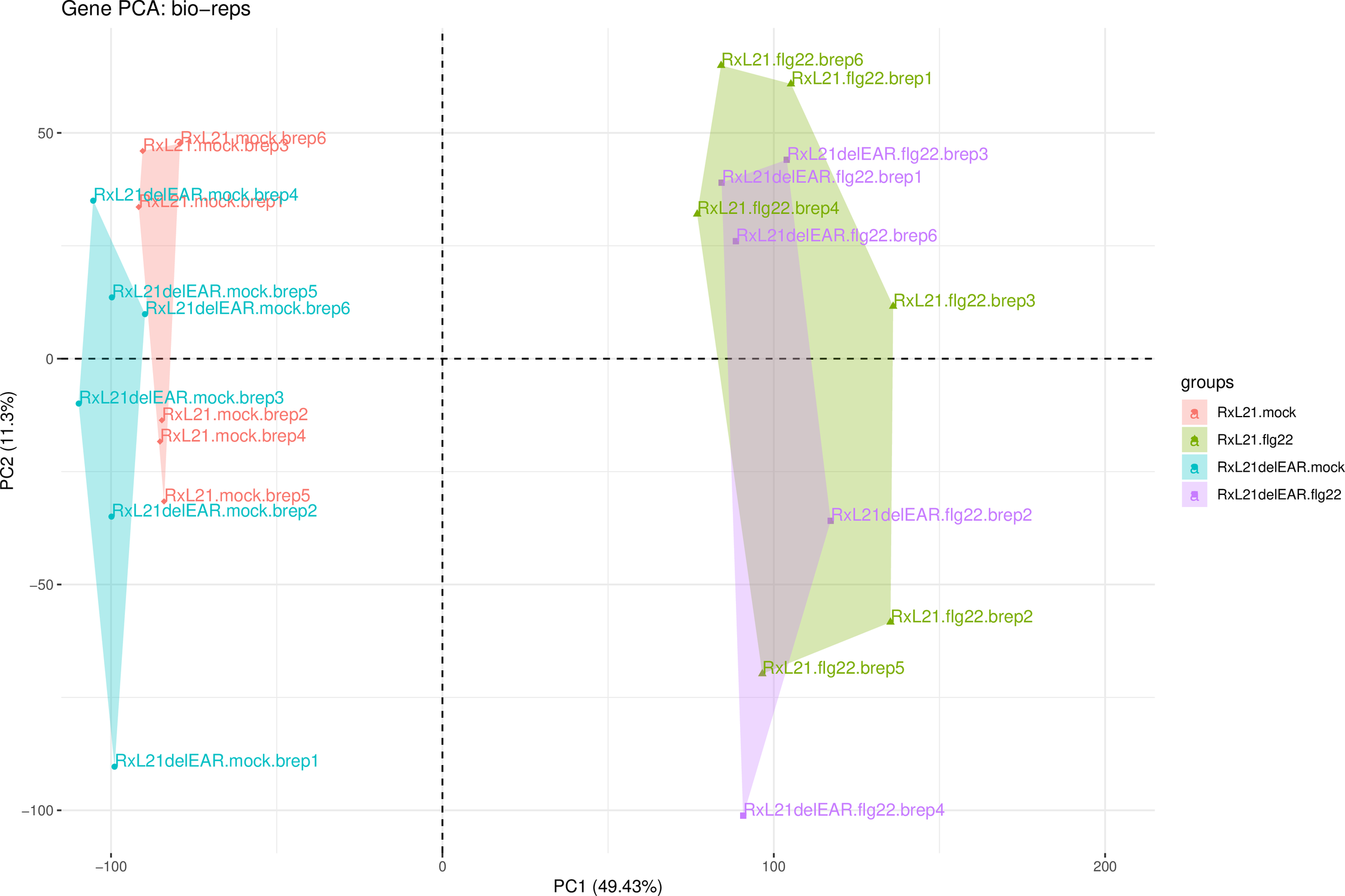

Supplement: S10 Fig — A principal component analysis (PCA) plot characterizes the trends shown by the RNAseq data. Each point represents a sample (mean of 2 technical replicates) and colour characterizes the sample group. Groups consist of 6 bioreps (3 samples from each of two independent transgenic Arabidopsis lines expressing 35S:HA:RxL21 or 35S:HA:RxL21ΔEAR). (TIF) [file ppat.1008835.s011.tif]

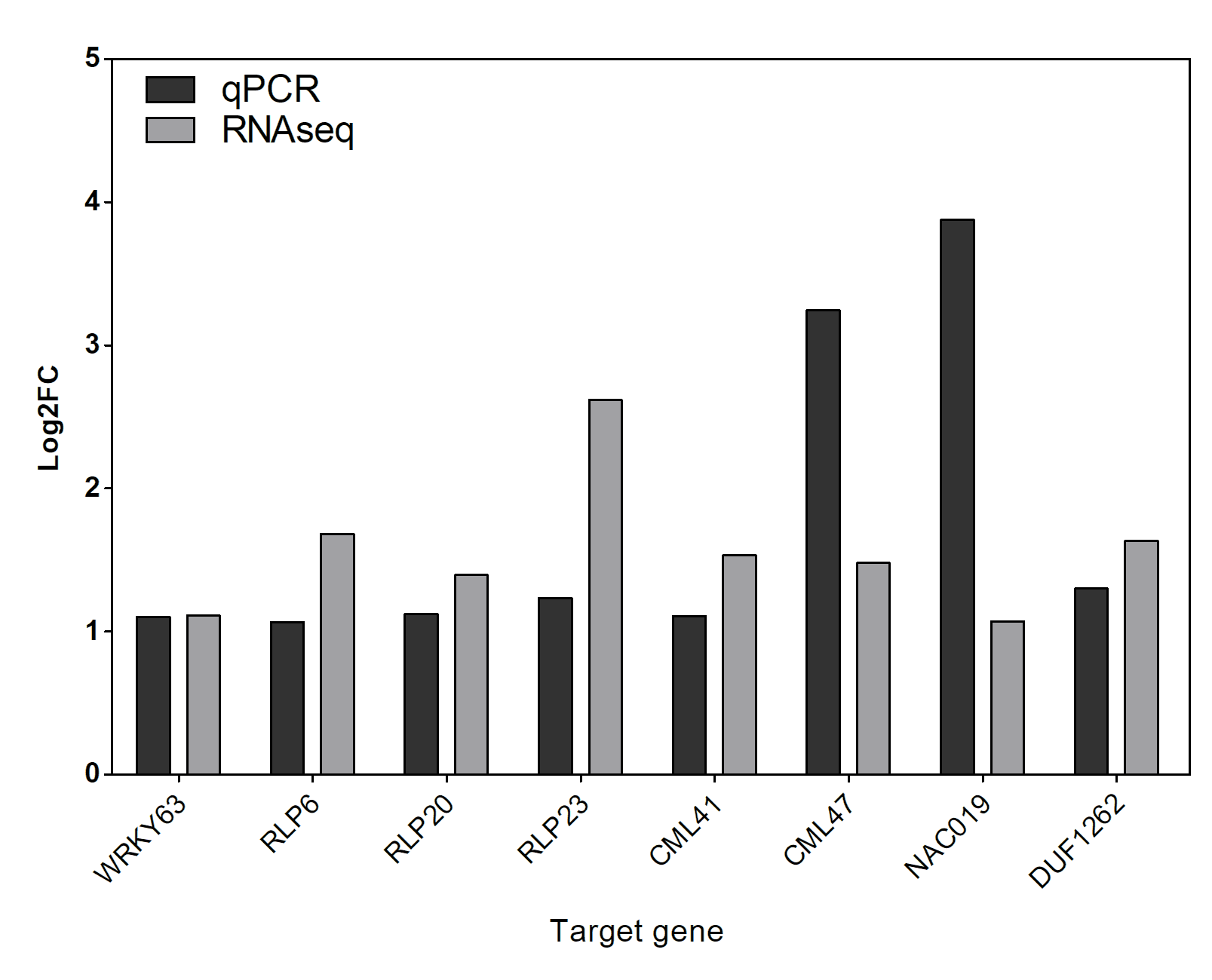

Supplement: S11 Fig — Comparison of log2 fold change (log2FC) for the 8 selected genes between RxL21 and RxL21ΔEAR lines by qPCR compared to RNA-seq read counts. Mean fold change of three biological replicates is shown. (TIF) [file ppat.1008835.s012.tif]
